# Tissue- Specific Expression Analysis of Anthocyanin Biosynthetic Genes in White- and Red-Fleshed Grape Cultivars

**DOI:** 10.3390/molecules201219883

**Published:** 2015-12-19

**Authors:** Sha Xie, Changzheng Song, Xingjie Wang, Meiying Liu, Zhenwen Zhang, Zhumei Xi

**Affiliations:** 1 College of Enology, Northwest A & F University, No. 22 Xinong Road, Yangling 712100, China; qdp.xie.sha@163.com (S.X.); scz0809@sina.com (C.S.); 18202991106@163.com (X.W.); qq493235763@163.com (M.L.); 2 Shaanxi Engineering Research Center for Viti-Viniculture, Yangling 712100, China

**Keywords:** anthocyanin, grapevine, tissue specificity, skin, flesh, gene expression, colour, *Vitis vinifera*

## Abstract

Yan73, a *teinturier* (dyer) grape variety in China, is one of the few *Vitis vinifera* cultivars with red-coloured berry flesh. To examine the tissue-specific expression of genes associated with berry colour in Yan73, we analysed the differential accumulation of anthocyanins in the skin and flesh tissues of two red-skinned grape varieties with either red (Yan73) or white flesh (Muscat Hamburg) based on HPLC-MS analysis, as well as the differential expression of 18 anthocyanin biosynthesis genes in both varieties by quantitative RT-PCR. The results revealed that the transcripts of GST, OMT, AM3, CHS3, UFGT, MYBA1, F3′5′H, F3H1 and LDOX were barely detectable in the white flesh of Muscat Hamburg. In particular, GST, OMT, AM3, CHS3 and F3H1 showed approximately 50-fold downregulation in the white flesh of Muscat Hamburg compared to the red flesh of Yan73. A correlation analysis between the accumulation of different types of anthocyanins and gene expression indicated that the cumulative expression of GST, F3′5′H, LDOX and MYBA1 was more closely associated with the acylated anthocyanins and the 3′5′-OH anthocyanins, while OMT and AM3 were more closely associated with the total anthocyanins and methoxylated anthocyanins. Therefore, the transcripts of OMT, AM3, GST, F3′5′H, LDOX and MYBA1 explained most of the variation in the amount and composition of anthocyanins in skin and flesh of Yan73. The data suggest that the specific localization of anthocyanins in the flesh tissue of Yan73 is most likely due to the tissue-specific expression of OMT, AM3, GST, F3′5′H, LDOX and MYBA1 in the flesh.

## 1. Introduction

Anthocyanins are a class of flavonoids that are responsible for the characteristic colour of many flowers, fruits, foliage, seeds, and other tissues [1]. In red and black grape berries, anthocyanins are the predominant pigment extracted during wine processing. Therefore, knowledge about the quantitative and qualitative patterns of anthocyanin accumulation in developing berries is critical for determining the wine colour. In the majority of grapevine varieties, anthocyanins accumulate only in the skin. However, there are also some red grape cultivars, known as *teinturier* (dyer) varieties, able to synthesize anthocyanins in the flesh [2].

Anthocyanins are synthesized on the cytoplasmic surface of the endoplasmic reticulum (ER) by a branch of the flavonoid pathway, and subsequently transported to the large vacuole for storage [3]. The anthocyanin biosynthetic pathway has been well characterized in plants, including *Vitis vinifera* [4]. Based on studies in numerous plant cultivars, it has been determined that anthocyanin biosynthesis is largely controlled at the transcriptional level and regulated by multi-protein complexes composed of R2R3-MYB domains, basic helix-loop-helix (bHLH) motifs or WD40 repeats [5,6]. However, anthocyanin accumulation is not widespread; rather it occurs in specific groups of cells located in the epidermal or cortical regions or close tovascular bundles [5]. Furthermore, the transcriptional regulation of anthocyanin biosynthesis is tissue- and species-specific [7]. In the case of apple flesh, the MdMYB110a_JP gene isolated from the apple cultivar “JPP35” by Umemura *et al.* is responsible for the red flesh but not the skin colour of the fruit [8]. Similarly, in studies on the differential expression of structural genes involved in anthocyanin biosynthesis among white-skinned grape cultivars, Kobayashi *et al.* have found that CHS expression was not detectable in Italia (*V. vinifera*) skin, and PAL expression was not detected in Muscat of Alexandria (*V. vinifera*) skin [9]. All the data strongly suggest that there are different mechanisms by which the anthocyanin biosynthetic pathway is regulated among various cultivars and tissues [10].

Like other fleshy fruits, grapevine berries are complex organs, formed by distinct tissues that exhibit different anthocyanin patterns [11,12]. The colour difference between red and white berry tissues is due to the presence or absence of anthocyanins in particular cell layers. In the last decades, grape skin colour has been more extensively studied from a molecular genetic point of view [13,14]. The phenotypic change from white- to red-skinned cultivars is thought to be the result of retrotransposon-induced mutations in VvmybA1, which blocks UFGT expression [15]. Based on transcriptomic analyses, differential regulation exists in the flesh and skin tissues of grape berries [16,17]; however, few studies have addressed gene expression patterns related to colour in grape flesh tissue. In fact, it has been previously documented that the expression of structural genes involved in anthocyanin biosynthesis (except DFR) was not detected in the flesh tissue of Shiraz grape [13]. Recently, Kobayashi *et al.* confirmed these findings in the flesh of the Kyoho grape and found that MYBA genes were markedly expressed in Kyoho flesh [18]. However, these findings have not been well elucidated. It is possible that recent studies concerning *teinturier* grape cultivars will pave the way for further investigation of the tissue-specific expression of anthocyanin biosynthetic genes [11,19]. *Teinturier* varieties, such as Alicante Bouschet, Petit Bouschet and Morrastrel Bouschet, were developed in the nineteenth century by Louis and Henri Bouschet [2]. At maturity, the berries of these varieties are entirely red, with anthocyanin biosynthesis in both the skin and the pulp. Moreover, anthocyanins begin to accumulate in the flesh before they accumulate in the skin of the Alicante Bouschet cultivar [12]. Several studies have indicated that there are significant differences in the quantity and quality of anthocyanins in the skin and flesh of Alicante Bouschet [20]. Furthermore, in a recent study on the various organs of Alicante Bouschet, Falginella *et al.* have suggested that anthocyanin biosynthesis exhibits discontinuous patterns in various berry tissues and that anthocyanin genes are spatially regulated [7].

Likewise, *Vitis vinifera* “Yan73”, a Chinses *teinturier* (dyer) variety, is able to accumulate anthocyanins in the skin, pulp, pedicels, and rachis [19]. This variety is obtained from a cross between Muscat Hamburg (*Vitis vinifera* L.) and Alicante Bouschet (*Vitis vinifera* L.) [21] and is commonly used for blending with pale red wines to increase their colour intensity. Until now, most of the studies concerning Yan73 have focused on the differences in anthocyanin profiles between the skin and flesh [19,21], but never on the molecular events. Due to anthocyanin accumulation in the flesh, Yan73 is an useful material for advancing our understanding of tissue-specific expression of genes involved in anthocyanin biosynthesis. Our study was performed to identify the tissue- specific expression of genes associated with berry colour in Yan73. Therefore, we compared the accumulation of anthocyanins in the skin and flesh tissues of two red-skinned grape varieties with either red (Yan73) or white flesh (Muscat Hamburg) based on HPLC, as well as the expression of 18 anthocyanin biosynthesis genes in both varieties by quantitative RT-PCR. In this study, Muscat Hamburg was selected as the control to identify genes whose expression may be related to berry colour and exclude genes associated with variety-dependent differences because Muscat Hamburg is the male parent of Yan73.

## 2. Results and Discussion 

### 2.1. Anthocyanin Profiling in the Skin and Flesh of Two Different V. vinifera Cultivars

We observed differences in the quantity and types of anthocyanins in the skin and flesh tissue of the two different cultivars (Table 1). The total anthocyanin content of mature Yan73 berry skin was higher than in the flesh, but the synthesis of anthocyanins occurred initially in the flesh and later in the skin, and the anthocyanin content of Yan73 skin exceeded that in the flesh until 92 days after anthesis (DAA). A similar result was observed in red-fleshed *V. vinifera* L. “Alicante Bouschet” [7]. Previous data have documented that there are two possibilities to explain why red plant pigments are accumulated in the flesh of Yan73: one is due to the tissue-specific expression of some genes involved in anthocyanin biosynthesis; the other is due to the direct transport of anthocyanin products [21]. Our results appear to support the first possibility. Because the anthocyanins are synthesized in the flesh before the skin of Yan73, it is not possible that the anthocyanins of skin are transported to the flesh of Yan73. The skin of Yan73 had a higher relative abundance of 3′5′-OH anthocyanins and acylated anthocyanins compared with the flesh throughout the period of anthocyanin synthesis, in agreement with a previous report [21]. On the other hand, both the skin and pulp of Yan73 had similar percentages of methoxylated anthocyanins and these percentages were sustained at a high level during all stages of fruit development.

The anthocyanins in Muscat Hamburg displayed quantitative and qualitative differences from those in Yan73, although Muscat Hamburg is the male parent of Yan73. Consistent with other red-skinned cultivars with white pulp (Gamay) [10], the flesh of Muscat Hamburg did not contain any detectable anthocyanins. The total anthocyanins and the proportions of 3′5′-OH anthocyanins and acylated anthocyanins in the skin of Muscat Hamburg were lower than those in Yan73 skin. These results suggest the complexity of the anthocyanin pathway and indicate that there may be differences in the mechanisms by which the pathway is controlled among different organs and plant species [10].

### 2.2. The Differential RNA Expression Profile of Anthocyanin Biosynthetic Enzymes in the Skin and Flesh of Two Different V. vinifera Cultivars

**Figure 1:**
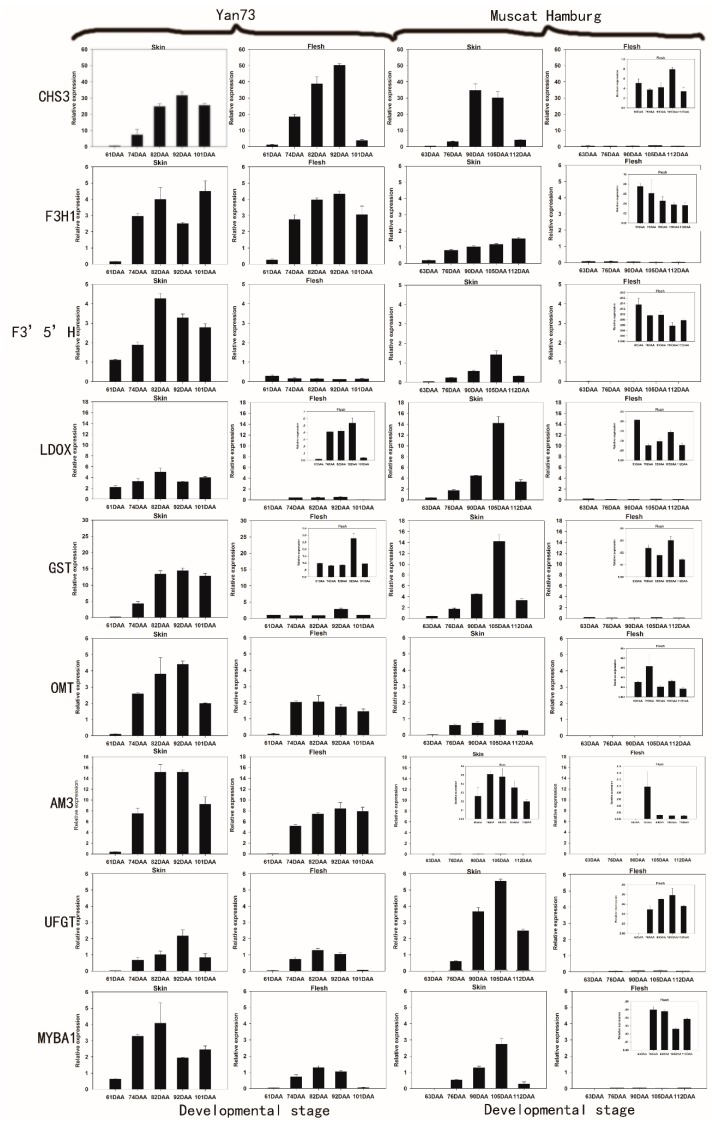
Expression patterns of anthocyanin biosynthetic genes in the skin and flesh of Yan 73 and Muscat Hamburg cultivars across five developmental stages. The data are derived from qPCR analysis and are normalized to *VvUbiquitin*. The transcriptional levels of the same gene in the skin and flesh tissues of Yan 73 and Muscat Hamburg are expressed on the y axes with the same scale, and relatively lower values are magnified in inset graphs with y axes with narrower scales. Berry developmental stage is referred to on the *x* axis. DAA, days after anthesis; 61 DAA (63 DAA) pre-véraison; 74 DAA (76 DAA), 50% véraison green; 82 DAA (90 DAA), 50% véraison red; 92 DAA (105 DAA), 100% véraison; 101 DAA (112 DAA) , harvest. Error bars illustrate the standard errors for three replicates.

To explore coordinated gene expression patterns related to colour determination in different grape berry tissues, we evaluated the levels of transcripts encoding 18 anthocyanin biosynthetic genes in both the skin and pulp of two red-skinned cultivars with either red (Yan73) or white flesh (Muscat Hamburg) by quantitative RT-PCR. Among the 18 genes, including early and late biosynthetic genes as well as transcription factors, GST, OMT, AM3, CHS3, UFGT, MYBA1, F3′5′H, F3H1 and LDOX were barely detectable in the non-pigmented pulp of Muscat Hamburg and were at least 10-fold less abundant in the white flesh of Muscat Hamburg compared to the red flesh of Yan73 (Figure 1). In particular, GST, OMT, AM3, CHS3 and F3H1 showed approximately 50-fold downregulation in the white flesh of Muscat Hamburg compared to the red flesh of Yan73. In 1996, Boss *et al.* reported that several flavonoid pathway genes (CHS, F3H and LDOX) were detected in all unpigmented tissues, including young leaves, tendrils, seeds, canes, roots and flowers, but were not detected in white flesh using northern analysis [13]. Similarly, in a recent study of Gamay berries, CHS3, GST and F3H1 were also nearly undetectable in white flesh by QPCR, whereas these genes were detectable in red skin [10]. These results suggest that the organ-specific expression of one or more anthocyanin biosynthetic genes might result in the differences in anthocyanin accumulation and colour among different tissues. In our study, GST, OMT, AM3, CHS3, UFGT, MYBA1, F3′5′H, F3H1 and LDOX were the candidate genes that were strongly associated with berry colour.

**Table 1:** Anthocyanin profiles of the skin and flesh tissues of Yan73 and Muscat Hamburg across 5 developmental stages.

DAA ^a^	Total Anthocyanins (mg/kg Fresh Berries) ^b^		%3′5′-OH Anthocyanins ^c^		3′5′-/3′-OH Anthocyanins ^d^		% Acylated Anthocyanins ^c^		%Methoxylated Anthocyanins ^c^	
Yan73	Skin	Flesh	Skin	Slesh	Skin	Flesh	Skin	Flesh	Skin	Flesh
61	6.95 ± 0.09a ^e^	6.54 ± 0.35a	89.17	72.33	8.23	2.61	70.80	33.43	97.36	92.82
74	66.58 ± 1.45a	92.07 ± 1.44b	86.66	72.36	6.50	2.62	36.82	29.83	95.61	96.40
82	410.53 ± 19.35b	479.56 ± 12.54c	87.18	17.63	6.80	0.21	36.82	20.07	94.37	95.22
92	1470.17 ± 28.19c	599.77 ± 27.54d	87.73	22.79	7.15	0.30	41.14	17.26	89.45	95.79
101	1646.05 ± 46.27d	650.60 ± 3.18d	87.50	21.73	7.00	0.28	41.25	19.73	90.19	95.54
Muscat Hamburg										
63	5.52 ± 0.24a	ND ^f^	13.60	ND	0.16	ND	19.79	ND	45.20	ND
76	80.91 ± 2.08b	ND	31.31	ND	0.46	ND	11.51	ND	88.60	ND
90	224.90 ± 6.18c	ND	32.42	ND	0.48	ND	11.91	ND	91.08	ND
105	419.63 ± 4.18d	ND	33.39	ND	0.50	ND	11.38	ND	90.86	ND
112	371.44 ± 5.55e	ND	43.61	ND	0.77	ND	32.14	ND	97.14	ND

^a^ DAA: Days after anthesis; ^b^ Values are expressed as the mean ± standard deviation of triplicate samples for total anthocyanins. Each anthocyanin concentration was calculated on the basis of fresh weight of the whole berries; ^c^ Values are expressed as the percentage of the total anthocyanins; ^d^ Values are expressed the ratio of 3′5′-OH anthocyanins to 3′-OH anthocyanins; ^e^ Different letters in the same column across 5 developmental stages indicate significant differences based on Tukey’s HSD test at *p* < 0.05; ^f^ ND: not detected.

Among the nine candidate genes, CHS3 and F3H1 were globally more highly expressed in the flesh than in the skin of Yan73, but both genes were almost undetectable in the white flesh of Muscat Hamburg. To date, three isoforms of CHS (CHS1,CHS2 and CHS3) have been isolated from Cabernet Sauvignon, but only CHS3 was correlated specifically with berry colour and found to accumulate mainly in the berry skin of red cultivars during colouration [10,22]. Our results appear to confirm these reports, as CHS3 showed the highest expression in red flesh, but was barely expressed in white flesh. Some reports suggested that F3H1 transcription coincided with anthocyanin synthesis [23,24]. However, Ageorges *et al.* found that F3H1 was not specifically expressed in pigmented berry tissues, as no significant differential expression between Pinot Noir and Pinot Blanc was observed [10]. However, in our study, red flesh displayed significantly higher levels of F3H1 transcription than white flesh, indicating that F3H1 might be specifically expressed in the red berry tissues. This result may have occurred because there are different mechanisms by which the anthocyanin pathway is controlled in different plant species and different tissues. 

In our study, both F3′5′H and LDOX showed at least 10-fold higher expression in the skin than in the flesh in bothYan73 and Muscat Hamburg. Thus, F3′5′H expression could explain why Yan73 skin synthesizes greater amounts of 3′5′-OH anthocyanins than the flesh. These results confirmed that variation in the anthocyanin profile between different tissues is strongly related to the expression of F3′5′H [25]. Regulation of F3′5′H is largely genotype-specific, and it is almost not expressed in green-skinned cultivars [25,26]. Similarly, F3′5′H transcription was absent in the white flesh of Muscat Hamburg in this study. LDOX was previously considered to be the key enzyme for anthocyanin synthesis in grapes, however, researchers later found that that it was expressed in non-pigmented tissues such as young leaves, tendrils, seeds, canes, roots and flowers [13]. In fact, LDOX was even expressed in the white skin of Italia and Muscat of Alexandria, although at a low level [9]. Additionally, in contrast to what was observed with UFGT, delivery of LDOX to the somatic embryos of grape failed to induce reddish-purple spots. [18]. Therefore, although LDOX expression was undetectable in white flesh in several studies, the major control point of anthocyanin biosynthesis was not thought to be LDOX [13].

The genes GST, OMT, AM3 and UFGT showed similar expression patterns in pigmented tissues and they were all highly expressed in Yan73 skin compared to flesh. A similar result was observed in a study of another *teinturier* variety (Alicante Bouschet) [7]. The expression of these genes commenced after the coloration stage (74DAA for Yan73 and 76DAA for Muscat Hamburg) and peaked between mid-véraison and full-véraison (82 or 92DAA for Yan73 and 105DAA for Muscat Hamburg). In addition, AM3 was expressed at a trace level in the Muscat Hamburg variety compared to Yan73, which indicated the plant-specificity of AM3 expression. This trace level of expression coincides with a lower percentage of acylated anthocyanins in Muscat Hamburg compared with Yan73, as the grapevine AM3 protein is a specific transporter of acylated anthocyanins [27]. GST, OMTand AM3 are thought to be important for anthocyanin biosynthesis and have been found to be involved in vacuolar anthocyanin transport in grapevines [7,28]. In our study, the expression patterns of GST, OMT and AM3 overlapped with each other and with that of UFGT. Furthermore, it should be noted that in the Yan73 cultivar, MYBA1 gene expression began earlier than the expression of GST, OMT, AM3 and UFGT genes in this study. Its expression could be detected before véraison (61DAA) and peaked in mid-véraison berries. These results seem to indicate that similar to UFGT, GST, OMT and AM3, are also target genes of the MYBA1 transcription factor. The same results were observed in grapevine hairy roots, in which ectopic expression of MYBA1 enhanced the overexpression of GST and OMT [28]. 

**Figure 2:**
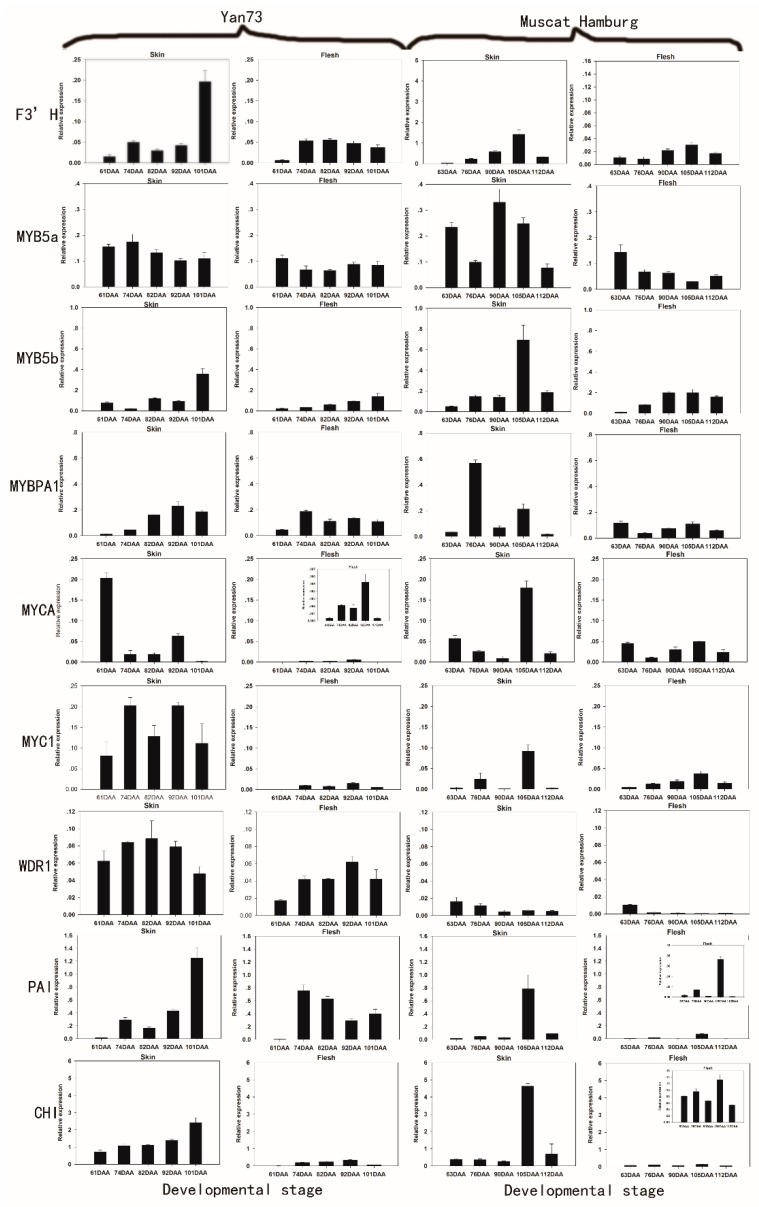
Expression patterns of anthocyanin biosynthetic genes in the skin and flesh of Yan 73 and Muscat Hamburg cultivars across five developmental stages. The data are derived from qPCR analysis and are normalized to *VvUbiquitin*. The transcriptional levels of the same gene in the skin and flesh tissues of Yan 73 and Muscat Hamburg are expressed on the *y* axes with the same scale, and relatively lower values are magnified in inset graphs with y axes with narrower scales. Berry developmental stage is referred to on the *x* axis. DAA, days after anthesis; 61 DAA (63 DAA) pre-véraison; 74 DAA (76 DAA), 50% véraison green; 82 DAA (90 DAA), 50% véraison red; 92 DAA (105 DAA), 100% véraison; 101 DAA (112 DAA), harvest. Error bars illustrate the standard errors for three replicates.

In contrast, the white flesh of Muscat Hamburg showed moderate levels of MYB5a, MYB5b, MYBPA1, MYCA, MYC1, F3’H and WDR1 expression (Figure 2), indicating that these genes may not be related specifically to berry colour but rather may be involved in the synthesis of other secondary metabolites [29]. 

In fact, numerous reports have established that these MYB transcription factors are involved in the control of various aspects of the flavonoid synthesis pathway: MYB5a and MYB5b are involved in various branches of the phenylpropanoid pathway, which leads to the synthesis of leading to flavonols, proanthocyanidins (PAs) and anthocyanins [30,31]; and MybPA1 is involved in the PA pathway [28]. MYCA, MYC1 and WDR1, as the bHLH and WDR protein families, participate in not only the control of phenylpropanoid biosynthesis but also the regulation of epidermal cell differentiation and cell patterning in root hair and trichome development [32]. F3′H is expressed in both anthocyanin-pigmented and green-skinned varieties, suggesting that F3’H could affect non-anthocyanin biosynthetic pathways [25]. 

In conclusion, among the 18 genes investigated in this study, the expression of GST, OMT, AM3, CHS3, UFGT, MYBA1, F3′5′H, F3H1 and LDOX was non-detectable in the white- flesh of Muscat Hamburg. In Yan73, all of these genes with the exception of CHS3 and F3H1 were more highly expressed in the skin than in the flesh, consistent with the relatively abundant anthocyanins in the skin compared to the flesh. Taken together, these data suggest that the tissue-specific expression of GST, OMT, AM3, CHS3, UFGT, MYBA1, F3′5′H, F3H1 and LDOX might result in the differences between white- and red-flesh.

### 2.3. Correlations between Gene Expression and Anthocyanin Profiles

**Figure 3:**
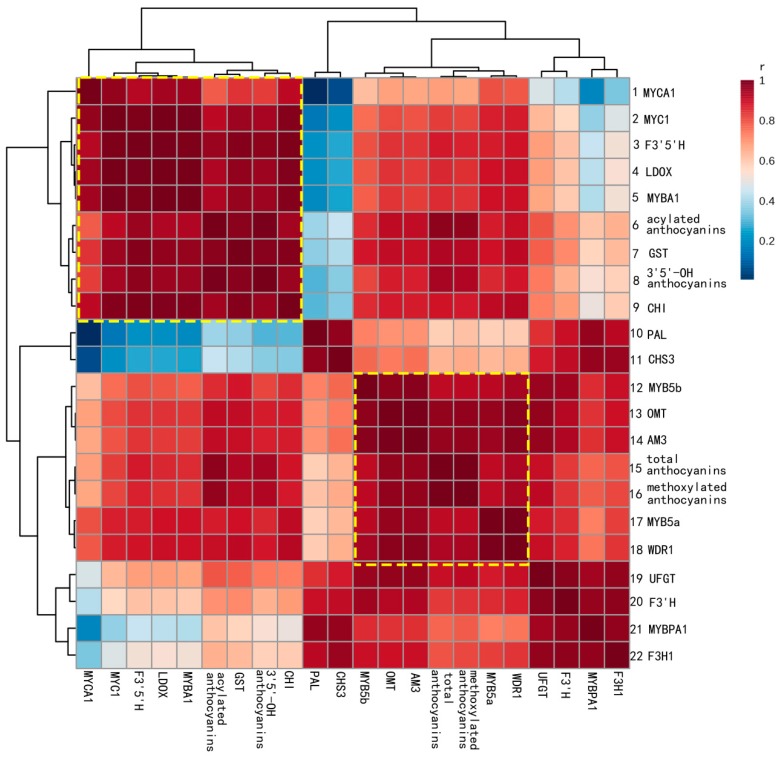
Heat map of the clustered correlations between anthocyanin accumulation patterns and gene expression in Yan73. Five sampling stages, three biological replicates, skin and flesh tissues were treated as independent factors in anthocyanin-transcript pairwise comparisons, which were carried out for 30 transcriptional data points for each gene. The correlation between anthocyanin accumulation and gene expression was calculated using Pearson’s correlation coefficient. The r and P values of the correlation are given in the Supplementary Materials (Table S1).

Yan73 was used as an example to explore the relationship between gene expression and anthocyanin profiles. For this purpose, the cumulative transcription of each gene from the onset of anthocyanin synthesis to harvest was calculated as the area below the expression curve of the gene throughout ripening (integral of gene expression) according to the literature [7,24]. Five sampling stages, three biological replicates, as well as skin and flesh tissues were treated as independent factors in anthocyanin-transcript pairwise comparisons. Thus, there are 30 transcriptional data points for each gene. The correlation analysis between accumulation of various types of anthocyanins and gene expression was performed using Pearson’s correlation coefficient, and the results are presented in Figure 3.

All candidate genes, except forCHS3 and F3H1, showed a strong correlation (*r* > 0.85) with the final anthocyanin content (Table S1). The heat map of the clustered correlations shows the major subsets of coexpression patterns (Figure 3). The set of genes in rows 1–9 was coexpressed with the acylated anthocyanins (AA) and the 3′5′-OH anthocyanins (OA). In particular, GST (*r =* 0.9760 for AA and *r =* 0.9803 for OA), F3′5′H (*r =* 0.9537 for AA and *r =* 0.9728 for OA,) LDOX (*r =* 0.9308 for AA and *r =* 0.9521 for OA), and MYBA1(*r =* 0.9275 for AA and *r =* 0.9500 for OA) were more highly correlated with both types of anthocyanins. Genes in rows 12–18 were tightly clustered with the total anthocyanin (TA) and methoxylated anthocyanin (MA) contents. Among these genes, OMT (*r =* 0.9635 for TA and *r =* 0.9677 for MA) and AM3 (*r =* 0.9610 for TA and *r =* 0.9651 for MA) displayed the strongest correlation with the TA and MA contens. Linear regression between the cumulative transcription of six genes (OMT, AM3, GST, F3’5’H, LDOX and MYBA1) and the corresponding anthocyanin contents are shown in Figure S1.

In this study, OMT, AM3, GST, F3′5′H, LDOX and MYBA1 transcripts showed strong correlations with different types of anthocyanins and explained most of the variation in the amount and composition of anthocyanins in skin and flesh of Yan73. In addition, these genes were absent in the white flesh of Muscat Hamburg. These data indicate that OMT, AM3, GST, F3′5′H, LDOX and MYBA1 might be candidate genes associated with the red colour of the Yan73 flesh 

## 3. Materials and Methods

### 3.1. Plant Material

Yan73 and Muscat Hamburg (*Vitis vinifera*) grape berries were collected from Chateau Changyu Verna, Shaanxi, China (108°73′ N; 34°33′ E; 600–700 m above sea level). Both cultivars have been planted in north–south-oriented rows since 2009, and trained to the Vertical Shoot-Positioned system. Vines of the Yan73 cultivar were spaced at 1.0 m × 2.5 m and vines of the Muscat Hamburg were spaced at 1.0 m × 3.0 m. Samples were collected at five stages spanning 7 weeks of berry development, and the maturation states of the grape berries were determined by average berry weight and soluble solids (Supplementary Materials, Figure S2). At each stage, three 100-berry samples of each variety were randomly collected from at least seven 10-clusters of 30 whole grapevines on both sides of the canopy. All samples were immediately frozen in liquid nitrogen and stored at −80 °C until processed. The samples were processed according to the method described by Falginella *et al.* [7]. Briefly, the skin, flesh, and seeds were separated from frozen berries with a scalpel, and the skin and flesh were ground to a powder in liquid nitrogen. Subsequently, the samples were divided into separate batches for anthocyanin extraction and RNA extraction. 

### 3.2. Extraction of Anthocyanins and HPLC-MS Analysis

Skin power or pulp powder (0.5 g) was immersed in methanol (10 mL) containing 2% acetic acid, ultrasonicated for 10 min, and then shaken on an orbital shaker (SHZ-88A, Taicang Experiment Equipment Factory, Jiangsu, China) at 130 rpm for 30 min at 25 °C. Subsequently, the homogenate was centrifuged at 8000 r/min for 5 min. The supernatant was collected and the precipitate was re-extracted three times following the above procedure. All extracts were combined and evaporated to dryness in a rotary evaporator (SENCO-R series; Shanghai Shensheng Biotech Co. Ltd., Shanghai, China) at 35 °C under vacuum. The residue was suspended in sample buffer (10 mL) containing a 9:1 (*v*/*v*) A:B mobile phase (phase A: 6% (*v*/*v*) acetonitrile containing 2% (*v*/*v*) formic acid, phase B: 54% (*v*/*v*) acetonitrile containing 2% (*v*/*v*) formic acid). Three independent extractions were carried out for either the skin or the pulp. The extracts were stored at −40 °C until analysis. Before injection, the extracts were filtered through 0.45 µm filters (cellulose acetate and nitrocellulose, CAN). 

An Agilent 1100 series LC-MSD trap VL (Agilent, Santa Clara, CA, USA) equipped with a G1379A degasser, G1312BA Quatpump, G1313A ALS autosampler, G1316A column, G1315A DAD, and reverse- phase column (Kromasil C18, 250 × 4.6 mm i.d., 5 μm particle size) was used to determine the individual anthocyanins of the extracts. The mobile phases were (A) 6% (*v*/*v*) acetonitrile containing 2% (*v*/*v*) formic acid, and (B) 54% (*v*/*v*) acetonitrile containing 2% (*v*/*v*) formic acid. A gradient consisting of solvent B was applied at a flow rate of 1.0 mL/min as follows: 10-25% B for 18 min, 25% B for 2 min, 25%–40% B for 10 min, 40%–70% B for 5 min, and 70%–100% B for 5 min. The injection volume was 30 µL, and the detection wavelength was 525 nm. The MS conditions were as follows: electrospray ionization (ESI) interface, positive ion model, 30 psi nebulizer pressure, 12 mL/min dry gas flow rate, 300 °C dry gas temperature, and scans at *m/z* 100–1500.

The identification of the different anthocyanins was performed by comparing their order of elution and retention time with those of standards (malvidin-3-glucoside, cyanidin glucoside, delphinidin glucoside, peonidin glucoside, petunidin glucoside, and pelargonidin glucoside). The weights of the molecular ion and the fragment ion were compared with those of standards and references [21,33,34]. Quantification of anthocyanins was performed using the external- standard method with commercial standards. Standard calibration curves of six non-acylated anthocyanins (malvidin-3-glucoside, cyaniding glucoside, delphinidin glucoside, peonidin glucoside, petunidin glucoside, and pelargonidin glucoside) were obtained by injecting of standard solutions under the same conditions used for the samples, over the range of concentrations observed. Thus, the concentrations of the other acylated anthocyanins were expressed in equivalents of the corresponding non-acylated anthocyanin. 

### 3.3. RNA Extraction and cDNA Synthesis

Two different RNA extraction protocols were utilized depending on the plant material. Total RNA was extracted from grape skin using the cetyltrimethylammonium bromide (CTAB)-based RNA extraction protocol described by Reid *et al.* [35]. Total RNA was extracted from grape flesh following a sodium dodecyl sulphate (SDS)-based procedure. This protocol, specific to the RNA extraction of grape flesh, has been previously validated by our group and is reported for the first time in this paper. Specifically, a fine power of flesh from each variety was added to a 2-mL RNase-free Eppendorf tube containing washing solvent (800 μL, 0.10 mol/L Tris-HCl, 0.35 mol/L sorbitol, 5 mmol/L EDTA, 100 g/L polyethylene glycol (PEG) 6000). Subsequently, a 2% β-mercaptoethanol solution was added and the tube was shaken vigorously. After incubation for 5 min at room temperature, the mixture was centrifuged at 8000 r/min for 5 min at 4 °C, the pellet was discarded and the supernatant was transferred to a new tube. This step allow for removal of the water from the flesh tissue, thus reducing the influence of its higher water content on the extraction of RNA. Thereafter, pre-warmed (80 °C) extraction buffer (850 μL, 100 mmol/L LiCl, 100 mmol/L Tris, 50 mmol/L ethylenediaminetetraacetic acid (EDTA), 40 g/L SDS, 30 g/L polyvinyl pyrrolidone (PVP)) was added to the supernatant, mixed with 5% β-mercaptoethanol, and shaken vigorously. After incubation for 15 min on ice, the mixture was centrifuged at 12,000 r/min for 15 min at 4 °C. The supernatant was collected, extracted with equal volumes of chloroform: isoamyl alcohol (24:1), mixed gently by inversion, incubated for 10 min on ice, and centrifuged at 12,000 r/min for 15 min at 4 °C. An equal volume of cold isopropanol was added to the isolated aqueous layer, mixed gently, and then the tube was stored at −20 °C for 2 h. After centrifugation at 12,000 r/min for 15 min at 4 °C, the supernatant was decanted and the nucleic acid pellet was suspended in a mixture of 2 mol/L LiCl and 50 mmol/L EDTA (600 µL). The nucleic acid pellets were collected again by centrifugation at 12,000 r/min for 15 min at 4 °C, washed with ice cold 70% EtOH, air dried, and dissolved in RNase-free ddH_2_O. A Nanodrop ND-1000 Spectrophotometer (Nanodrop Technologies, Rockland, DE, USA) was used to determine the concentration and purity of the isolated RNA. RNA integrity analysis was performed by electrophoresis on a 1% agarose gel stained with ethidium bromide. The cDNA was synthesized from 2 µg of RNA using a PrimeScript™ RT reagent kit with gDNA Eraser (Takara, Kyoto, Japan). Prior to reverse transcription, gDNA Eraser was used to remove contaminating DNA according to the manufacturer’s instructions. 

### 3.4. Real Time Quantitative PCR

To determine the transcript levels of anthocyanin biosynthetic genes, quantitative PCR was carried out using an iQ™5 Multicolor Real-Time PCR Detection System (Bio-Rad Laboratories, Berkeley, CA, USA), with SYBR^®^ Premix Ex Taq™ (Takara). Each reaction (25 µL) contained 12.5 µL of SYBR^®^ Premix Ex Taq (Tli RNaseH Plus) (2×), 2.0 µL of cDNA (<100 ng) and 0.5 µL of each primer (10 µM). The two-step PCR procedure recommended in the manufacturers’ manual was performed: an initial denaturation at 95 °C for 30 s, followed by 40 cycles of 95 °C for 5 s, an annealing temperature specific to each primer pair (Supplementary Materials, Table S2) for 30 s, and a melting curve from 60 °C to 95 °C. The sequences of the primers for real time PCR are given in Table S2 (Supplementary Materials) and the melt curves of 19 pairs of primers are provided in Figure S3 (Supplementary Materials). The data were analysed using iQ™5 Optical System Software (version 2.1, Bio-Rad). Gene expression levels were normalized to Vv*Ubiquitin* expression and the relative expression levels of each gene were calculated with the 2^−ΔCT^ method, where ΔCT = CTtarget − CTVv*Ubiquitin*. 

### 3.5. Statistical Analysis

SPSS 20.0 (IBM, Armonk, NY, USA) was used to analyse the statistical parameters of the total anthocyanins. One-way analysis of variance (ANOVA) and Tukey’s HSD test were conducted to detect significant differences in the total anthocyanins of different developmental stages. Statistical analysis of gene expression patterns was performed using SigmaPlot software (version 12.0, Systat Software, Inc., Washington, DC, USA). Correlation analysis between accumulation of various types of anthocyanins and gene expression was performed using Pearson’s correlation coefficient. MetaboAnalyst 3.0 [36] was used to perform the correlation analysis [37]. 

## 4. Conclusions 

To our knowledge, this is the first report describing the tissue-specific expression patterns of anthocyanin biosynthetic genes in the flesh and skin of Yan73. Our results revealed that GST, OMT, AM3, CHS3, UFGT, MYBA1, F3′5′H, F3H1 and LDOX transcripts were absent in the white pulp from Muscat Hamburg and were at least 10-fold less abundant in white flesh of Muscat Hamburg compared to the red flesh of Yan73. In particular, the expression of GST, OMT, AM3, CHS3 and F3H1 showed approximately 50-fold downregulation in the white flesh of Muscat Hamburg compared to in the red flesh of Yan73. Among the 9 genes (GST, OMT, AM3, CHS3, UFGT, MYBA1, F3′5′H, F3H1 and LDOX), all except CHS3 and F3H1 showed higher expression in the skin than in the flesh of Yan73, which is consistent with the relatively greater abundance of anthocyanins in the skin compared to the flesh of Yan73. The correlation analysis between the accumulation of various types of anthocyanins and gene expression indicated that the cumulative expression of GST, F3′5′H, LDOX and MYBA1 was more closely associated with the acylated anthocyanin and 3′5′-OH anthocyanin conthents, while OMT and AM3 were more closely associated with the total anthocyanin and methoxylated anthocyanin content. Thus, variations in OMT, AM3, GST, F3′5′H, LDOX and MYBA1 transcripts explained most of the differences in the quantity and composition of anthocyanins in the skin and flesh tissues of Yan73. Taken together, these data suggest that the tissue-specific expression of OMT, AM3, GST, F3′5′H, LDOX and MYBA1 might result in the specific localization of anthocyanins in the flesh tissue of Yan73. Certainly, further investigation is required to elucidate the specific molecular mechanisms underlying anthocyanin accumulation in red flesh tissue. Moreover, Yan73 may become the archetype for studying tissue-specific expression and regulation of the genes involved in anthocyanin biosynthesis.

## Supplementary Materials

Supplementary materials can be accessed at: http://www.mdpi.com/1420-3049/20/12/19883/s1.
