# Supplementary material for: Tissue- Specific Expression Analysis of Anthocyanin Biosynthetic Genes in White- and Red-Fleshed Grape Cultivars
Source: Molecules. 2015 Dec 19;20(12):22767–80. doi: 10.3390/molecules201219883 (PMC6331842; doi:10.3390/molecules201219883)
Supplement: Supplementary file 1 [file molecules-20-19883-s001.pdf]

Supplementary Materials: Tissue- Specific Expression Analysis of Anthocyanin Biosynthetic Genes in White- and Red-Fleshed Grape Cultivars

Sha Xie, Changzheng Song, Xingjie Wang, Meiying Liu, Zhenwen Zhang and Zhumei Xi

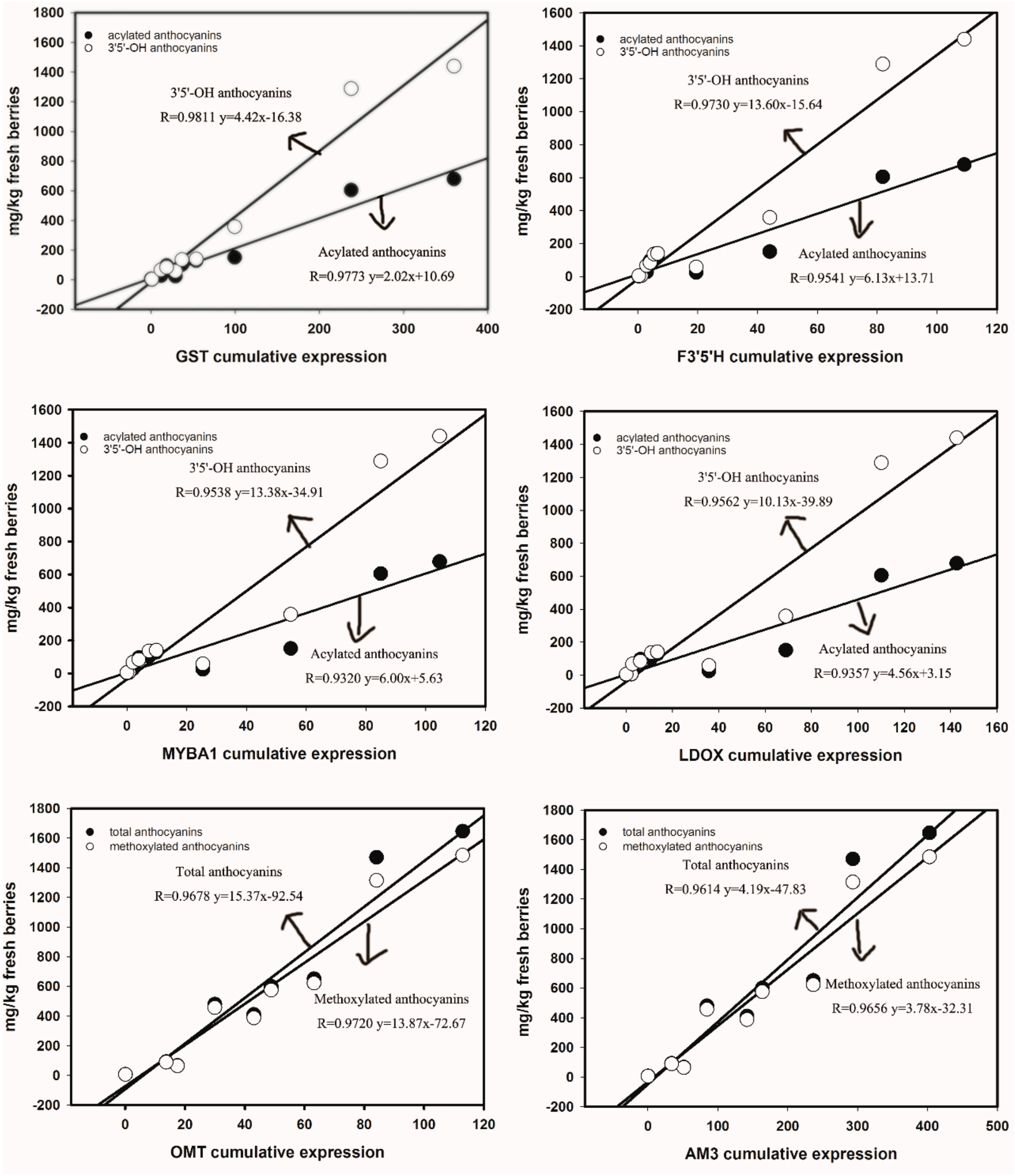

Figure S1. Linear regression between the cumulative transcription of genes (OMT, AM3, GST, F3′5′H, LDOX and MYBA1) and the accumulation of different types of anthocyanins.

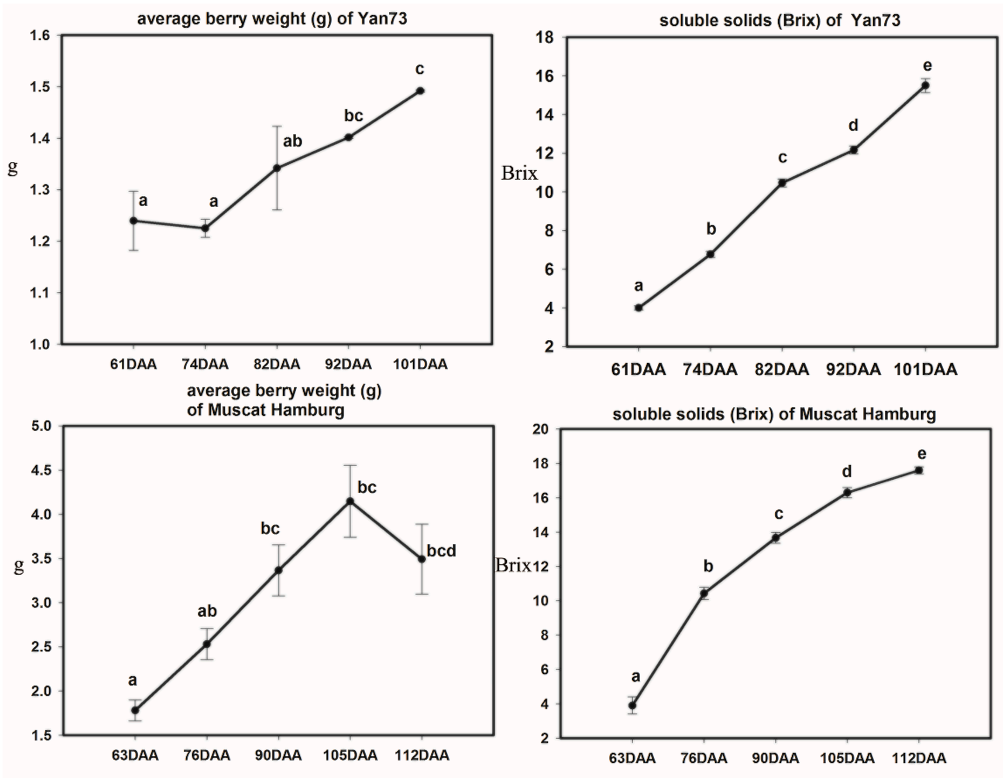

Figure S2. The progression of growth and sugar accumulation of berries from Yan73 and Muscat Hamburg. Error bars represent standard errors for three replicates and different letters indicate significant differences based on Tukey's HSD test at $p < 0.05$. Berry developmental stage is presented on the x axis: DAA, days after anthesis; 61 DAA (63 DAA), pre-véraison; 74 DAA (76 DAA), 50% véraison green; 82 DAA (90 DAA), 50% véraison red; 92 DAA (105 DAA), 100% véraison; 101 DAA (112 DAA), harvest.

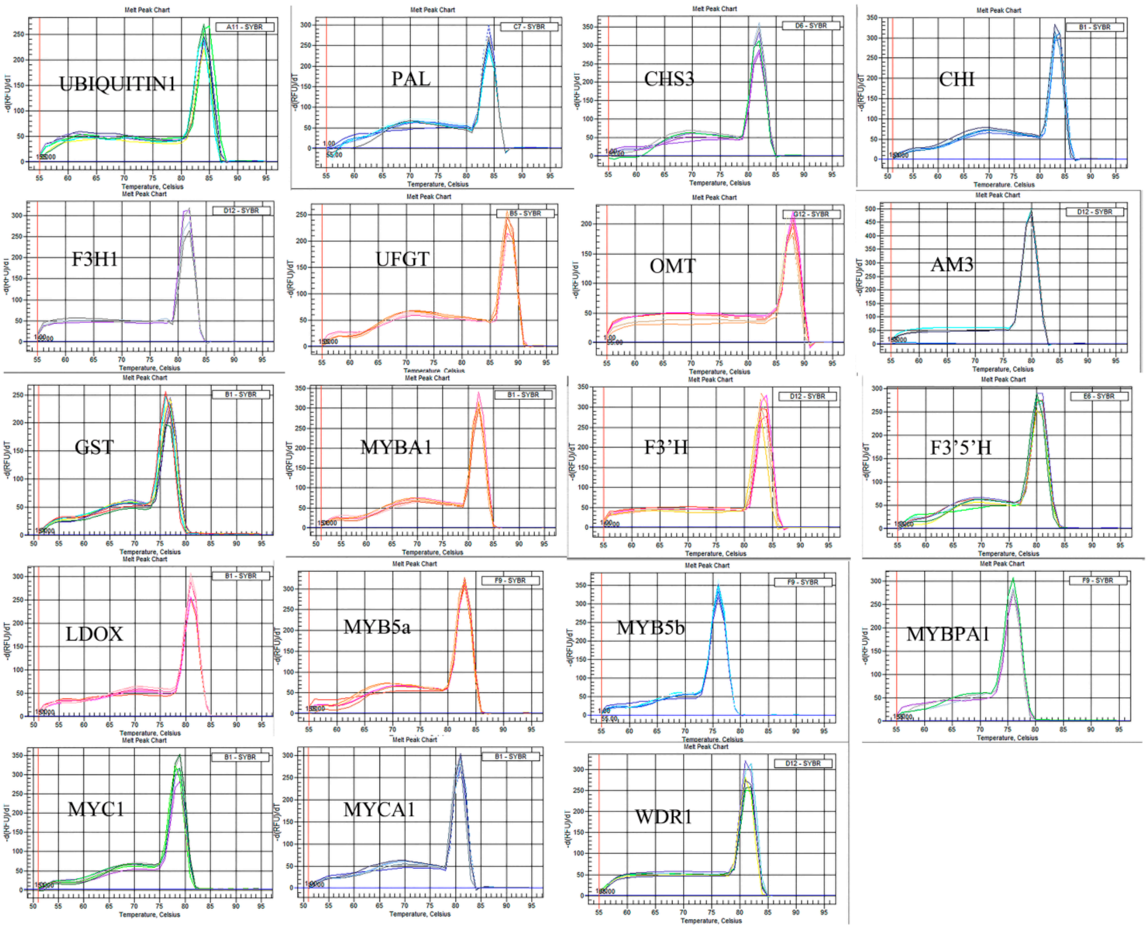

Figure S3. The melt peak of 18 anthocyanin biosynthetic genes.

Table S1. The *r* and *p* values of the correlations between anthocyanin accumulation patterns and gene expression in Yan73.

	**Total Anthocyanins**		**Methoxylated Anthocyanins**		**Acylated Anthocyanins**		**3′5′-OH Anthocyanins**	
	r	*P*	*r*	*p*	*r*	*p*	*r*	*p*
UFGT	**0.9023**	0.0003	**0.9123**	0.0002	0.7915	0.0055	0.7369	0.0139
PAL	0.5883	0.0719	0.6097	0.0597	0.388	0.265	0.3002	0.3978
CHS3	0.6362	0.048	0.6559	0.0394	0.4443	0.1981	0.3611	0.3056
F3′5′H	0.8774	0.0008	0.8672	0.0012	**0.9537**	<0.0001	**0.9728**	<0.0001
MYB5a	**0.9072**	0.0003	**0.9100**	0.0002	0.8758	0.0008	0.8578	0.0014
MYB5b	**0.9093**	0.0003	**0.9143**	0.0002	0.8528	0.0017	0.8164	0.0039
MYBPA1	0.7704	0.0091	0.7866	0.0069	0.6069	0.0626	0.5293	0.1156
LDOX	0.8573	0.0014	0.8479	0.0018	**0.9308**	0.0001	**0.9521**	<0.0001
CHI	0.8851	0.0007	0.8776	0.0008	**0.9427**	<0.0001	**0.9547**	<0.0001
MYC1	0.8257	0.0032	0.8776	0.004	**0.9116**	0.0002	**0.9383**	0.0001
MYBA1	0.8534	0.0016	0.8439	0.002	**0.9275**	0.0001	**0.9500**	<0.0001
GST	**0.9294**	0.0001	**0.922**	0.0001	**0.9760**	<0.0001	**0.9803**	<0.0001
MYCA1	0.6787	0.0306	0.6664	0.035	0.7866	0.0067	0.8269	0.0031
F3'H	0.8299	0.003	0.8432	0.0022	0.7038	0.023	0.6433	0.0448
OMT	**0.9635**	<0.0001	**0.9677**	<0.0001	**0.9071**	0.0002	0.8736	0.0008
AM3	**0.9610**	<0.0001	**0.9651**	<0.0001	**0.9056**	0.0003	0.8709	0.001
WDR1	**0.9323**	0.0001	**0.9345**	0.0001	**0.9015**	0.0004	0.8816	0.0007
F3H1	0.7951	0.0058	0.8106	0.0043	0.6441	0.0437	0.5772	0.0802

Table S2. Primer used for quantification of transcripts by real time quantitative PCR.

Gene	Encoded Protein	Forward Primer (5′-3′)	Reverse primer (5′-3′)	Reference
PAL	Phenylalanine ammonia-lyase	GCAGATTGGGAGAGGAGCA	CCGAACCGAATCAAGGAGT	[1]
CHS3	Chalcone synthase	TCGGCTGAGGAAGGGCTGAA	GGCAAGTAAAGTGGAAACAG	[2]
CHI	Chalcone isomerase	CAGGCAACTCCATTCTTTTC	TTCTCTATCACTGCATTCCC	[2]
F3H1	Flavanone 3-hydroxylase	CCAATCATAGCAGACTGTCC	TCAGAGGATACACGGTTGCC	[3]
UFGT	UDP-glucose-flavonoid-3-*O*-glucosyltransferase	GGGATGGTAATGGCTGTGG	ACATGGGTGGAGAGTGAGTT	[2]
OMT	*O*-methyltransferase	CTCTGCAGGCGCCTCTATTA	CCCAAAACAGAGTCTGGACA	[4]
AM3	AnthoMATE transporter3	GCAAACAACAGAGAGGATGC	AGACCTCGACAATGATCTTA	[5]
GST	Glutathione S-transferase	CGAGGGCGATTGTGAGGTA	TTCCACTTCCAGCCATTGAT	[1]
MYBA1	R2R3 Myb-type transcription factor	TAGTCACCACTTCAAAAAGG	GAATGTGTTTGGGGTTTATC	[2]
F3′H	Flavanone 3′-hydroxylase	ATGGTIGTIGARATGATG	CCRTAIGCYTCYTCCATRTT	[6]
F3′5′H	Flavanone 3′5-hydroxylase	AAACCGCTCAGACCAAAACC	ACTAAGCCACAGGAAACTAA	[7]
LDOX	Leucoanthocyanidin dioxygenase	AGGGAAGGGAAAACAAGTAG	ACTCTTTGGGGATTGACTGG	[2]
MYB5a	R2R3 Myb-type transcription factor	GTGCAGCAGCCATCTAATGTG	GCAGCAGGTTCCCAGACAGT	[7]
MYB5b	R2R3 Myb-type transcription factor	GGTGTTCTTTAATTTGGCTTCA	CACAACAACACAACCACATACA	[7]
MYBPA1	R2R3 Myb-type transcription factor	AGATCAACTGGTTATGCTTGCT	AACACAAATGTACATCGCACAC	[7]
MYC1	Basic helix-loop-helix transcription factor	GGAAGTAAAGAGGGCAATAAA	CTACAACATCAGCAACAATACCATA	[8]
MYCA1	Basic helix-loop-helix transcription factor	GAACAGGAGGGGATGAGTGA	CTTGGGAAGCACCTCCATTA	[9]
WDR1	WD-40 repeat protein	GCATTCTGAGGGAGATGGTC	TCCGAATCAAGAACCAAAGC	[9]
UBIQUITIN1	*Ubiquitin*	GTGGTATTATTGAGCCATCCTT	AACCTCCAATCCAGTCATCTAC	[4]

References

1. Conn, S.; Curtin, C.; Bezier, A.; Franco, C.; Zhang, W. Purification, molecular cloning, and characterization of glutathione *S*-transferases (GSTs) from pigmented *Vitis vinifera* L. cell suspension cultures as putative anthocyanin transport proteins. *J. Exp. Bot.* **2008**, *59*, 3621–3634.
2. Jeong, S.T.; Goto-Yamamoto, N.; Kobayashi, S.; Esaka, M. Effects of plant hormones and shading on the accumulation of anthocyanins and the expression of anthocyanin biosynthetic genes in grape berry skins. *Plant Sci.* **2004**, *167*, 247–252.
3. Ageorges, A.; Fernandez, L.; Vialet, S.; Merdinoglu, D.; Terrier, N.; Romieu, C. Four specific isogenes of the anthocyanin metabolic pathway are systematically co-expressed with the red colour of grape berries. *Plant Sci.* **2006**, *170*, 372–383.
4. Falginella, L.; di Gaspero, G.; Castellarin, S.D. Expression of flavonoid genes in the red grape berry of "Alicante Bouschet" varies with the histological distribution of anthocyanins and their chemical composition. *Planta* **2012**, *236*, 1037–1051.
5. Gomez, C.; Terrier, N.; Torregrosa, L.; Vialet, S.; Fournier-Level, A.; Verries, C.; Souquet, J.M.; Mazauric, J.P.; Klein, M.; Cheynier, V.; Ageorges, A. Grapevine MATE-type proteins act as vacuolar H^+-dependent acylated anthocyanin transporters. *Plant Physiol.* **2009**, *150*, 402–415.
6. Bogs, J.; Ebadi, A.; McDavid, D.; Robinson, S.P. Identification of the flavonoid hydroxylases from grapevine and their regulation during fruit development. *Plant Physiol.* **2006**, *140*, 279–291.
7. Azuma, A.; Yakushiji, H.; Koshita, Y.; Kobayashi, S. Flavonoid biosynthesis-related genes in grape skin are differentially regulated by temperature and light conditions. *Planta* **2012**, *236*, 1067–1080.
8. Hichri, I.; Heppel, S.C.; Pillet, J.; Leon, C.; Czemmel, S.; Delrot, S.; Lauvergeat, V.; Bogs, J. The basic helix-loop-helix transcription factor MYC1 is involved in the regulation of the flavonoid biosynthesis pathway in grapevine. *Mol. Plant* **2010**, *3*, 509–523.
9. Matus, J.T.; Loyola, R.; Vega, A.; Pena-Neira, A.; Bordeu, E.; Arce-Johnson, P.; Alcalde, J.A. Post-veraison sunlight exposure induces MYB-mediated transcriptional regulation of anthocyanin and flavonol synthesis in berry skins of *Vitis vinifera*. *J. Exp. Bot.* **2009**, *60*, 853–867.
